# Glucosylsphingosine (Lyso-Gb_1_): An Informative Biomarker in the Clinical Monitoring of Patients with Gaucher Disease

**DOI:** 10.3390/ijms232314938

**Published:** 2022-11-29

**Authors:** Matthew M. Gayed, Seung-Hye Jung, Erin Huggins, Eleanor Rodriguez-Rassi, Stephanie DeArmey, Priya Sunil Kishnani, Ashlee R. Stiles

**Affiliations:** 1 Division of Medical Genetics, Department of Pediatrics, Duke University Medical Center, Durham, NC 27710, USA; 2 Biochemical Genetics Laboratory, Duke University Health System, Durham, NC 27713, USA

**Keywords:** glucosylsphingosine, lyso-Gb_1_, chitotriosidase, Gaucher disease, biomarkers, long-term treatment

## Abstract

Historically, disease burden and treatment responses in patients with Gaucher disease (GD) was assessed by monitoring clinical data, laboratory, imaging, chitotriosidase (CHITO), and other biomarkers; however, these biomarkers lack specificity and CHITO is uninformative in patients heterozygous or homozygous for the *CHIT1* c.1049_1072dup24 variant. Recently, glucosylsphingosine (lyso-Gb_1_), a sensitive and specific GD biomarker, has been recommended for patient monitoring. Furthermore, studies measuring lyso-Gb_1_ and CHITO in patients on long-term treatment with enzyme replacement therapy (ERT) and/or substrate reduction therapy (SRT) reported as group data show a reduction in both analytes, yet individualized patient data are generally unavailable. We describe seven patients on long-term treatment with longitudinal clinical data with monitoring based on current treatment guidelines. We present four patients who exhibit stable disease with normalized CHITO despite elevated lyso-Gb_1_. We present one patient who transitioned from ERT to SRT due to lack of a clinical response with life-threatening thrombocytopenia who responded with marked improvement in platelets, and normalized levels of both CHITO and lyso-Gb_1_. Finally, we present two ERT to SRT switch patients with stable disease on ERT who exhibited non-compliance on SRT, one with mirrored marked elevations of CHITO and lyso-Gb_1_; and another with normal CHITO and platelets, but increasing lyso-Gb_1_ levels and enlarged spleen. These clinical vignettes highlight the role of lyso-Gb_1_ as a sensitive biomarker in management of patients with GD, and its further value when CHITO is normal and thus uninformative. We highlight the personalized medicine approach needed to optimize treatment outcomes and recommendations for these patients.

## 1. Introduction

Gaucher disease (GD) is an autosomal recessive lysosomal storage disorder caused by biallelic pathogenic variants in *GBA* and enzymatic deficiency of β-glucocerebrosidase. This enzymatic deficiency results in the accumulation of glucosylceramide in macrophages of the reticuloendothelial system, specifically the spleen, liver, lungs, and bone marrow. Involvement of these systems results in a wide range of symptoms including hepatosplenomegaly, thrombocytopenia, anemia, bone pain, bone crises, and avascular necrosis. The phenotypic manifestations of GD are clinically heterogeneous and disease subtype can be distinguished by age of onset, disease progression, extent of neurological involvement, and established genotype/phenotype correlations. Patients with type 1 GD, the most common subtype in Europe and North America [1] typically lack neurological involvement that is noted in Gaucher types 2 and 3 [2,3]. The visceral manifestations of GD are currently treated with either enzyme replacement therapy (ERT; imiglucerase, taliglucerase alfa, velaglucerase alfa) or substrate reduction therapy (SRT; eligustat, miglustat).

Historically, disease burden in patients with GD was first assessed by monitoring plasma levels of chitotriosidase (CHITO), an enzyme produced and secreted by activated macrophages [4,5]. While plasma CHITO activity is about 1000-fold elevated in untreated GD, its overexpression is not specific to GD as CHITO activity is elevated in other sphingolipidoses [4,5,6] and in patients with sarcoidosis and other interstitial lung diseases [7]. Furthermore, analysis is unreliable in approximately 33% of the general population who are heterozygous and 6% of the population who are homozygous for the *CHIT1* c.1049_1072dup24 (dup24) null variant in exon 10 [8], resulting in 50% reduced or complete absence of CHITO activity. Despite the lack of specificity, CHITO activity has been shown to correlate with liver and spleen volume, platelet counts [9,10] and more recently, with glucosylsphingosine (lyso-Gb_1_), a marker of glucosylceramide substrate accumulation [11]. In lysosomes, acid ceramidase actively converts glucosylceramide into lyso-Gb_1_, one of several adaptations in glucosylceramide metabolism [12]. Lyso-Gb_1_ is sensitive and specific to GD, elevated in 100% of treatment-naïve patients and shown to be an effective biomarker in treatment monitoring with significant correlation to CHITO activity, liver and spleen volumes, and treatment mode [13,14,15,16,17,18], and not noted in other conditions.

Studies measuring lyso-Gb_1_ and CHITO activity in patients on long-term treatment with ERT and/or SRT have shown that both analytes decrease comparably in the majority of patients [13,15,19], yet rarely normalize [20]. CHITO activity has been shown to decrease by half after two years of treatment with ERT and by 5 years of ERT remains about twice the upper limit of the normal reference range [13]. Similarly, lyso-Gb_1_ levels have been shown to decrease by half in the first year of treatment with ERT before reaching a plateau after 3–4 years of treatment with ERT with levels approximately 13.5-fold greater than the reference range [13]. In addition, its clinical utility has been observed in group data of ERT switch patients from imiglucerase to velaglucerase alfa, which noted further reduction in lyso-Gb_1_ levels after the switch [14]. Furthermore, SRT-treated patients with eliglustat observed an even greater decrease in lyso-Gb_1_ levels in comparison to ERT-treated patients [21] and similarly, in ERT to SRT switch patients, further decreases of CHITO and lyso-Gb_1_ were observed [22]. In treatment naïve patients, reduced levels of CHITO activity and lyso-Gb_1_ were noted after two years of treatment with ERT and/or SRT; however, normalization of neither CHITO activity nor lyso-Gb_1_ was achieved [21] in this cohort. Overall, from these data available, normalization of CHITO activity and lyso-Gb_1_ is rarely achieved. While one ERT to SRT switch patient has been reported with normalized levels of lyso-Gb_1_ after eliglustat treatment [14], additional individual patient data is currently lacking.

We present seven patients with GD with long-term clinical data and biomarker analysis measuring lyso-Gb_1_ levels and CHITO activity and describe different case scenarios showing the value of biomarker analysis, especially lyso-Gb_1,_ in regard to treatment outcomes and treatment recommendations. We present four patients (three treatment switch patients) who exhibit stable disease on treatment with now-normalized CHITO and platelets, persistent splenomegaly, and elevated lyso-Gb_1_. We present one switch patient on long-term treatment with ERT who transitioned to SRT and responded with a very good improvement with platelets gradually approaching and reaching the normal reference range, and now-normalized levels of both CHITO activity and lyso-Gb_1_. Furthermore, we present two switch patients on SRT who exhibit non-compliance, one with stable disease on long-term ERT with marked elevations of lyso-Gb_1_ and CHITO activity after transitioning to SRT; and another on SRT who self-reports missing doses with normal CHITO activity and platelets, but increasing levels of lyso-Gb_1_ and splenomegaly. These clinical vignettes highlight the role of biomarker analysis in monitoring patients with GD and demonstrate the utility of lyso-Gb_1_ as a sensitive biomarker when CHITO activity is otherwise uninformative.

## 2. Case Presentation

### 2.1. Methods

A retrospective chart review of seven patients with GD, six with type 1 GD and one patient with type 3 GD, followed at the Duke University Medical Center (DUMC) Metabolic Genetics clinic was conducted. All cases were followed and managed utilizing standard surveillance [23,24] and treatment guidelines [25,26]. Eliglustat dosage was prescribed as recommended with a dose regimen of 84 mg twice daily (BID), for patients who were found to be intermediate or extensive CYP2D6 metabolizers, and 84 mg once daily for patients who were found to be poor CYP2D6 metabolizers via pharmacogenetic testing. Switch patients are defined as a patient who has switched treatments, either from one ERT to another, from ERT to SRT, or a combination of the two (ERT to ERT to SRT).

Clinical history was obtained which included complete blood count, spleen volume, liver volume, bone density studies, and biomarker analysis which included CHITO and more recently, lyso-Gb_1_. Lyso-Gb_1_ analysis was performed according to a previously published method [27] by DUHS Biochemical Genetics Laboratory. CHITO analysis was a send-out test to LabCorp and Genzyme Laboratories. The normal reference range for lyso-Gb_1_ is ≤1.9 nmol/L. For the purpose of this manuscript, the normal reference range was defined as 4–120 nmol/h/mL for CHITO activity and 150–450 × 10^9^ cells/L for platelets. Liver and spleen volumes were collected from magnetic resonance imaging (MRI) and described as multiples of normal (MN). In order to calculate MN for hepatic and splenic volumes, normal spleen and liver values were first calculated based on previously established ratios. Normal splenic volume was assumed to be 0.2 percent of body weight in grams. Normal hepatic volumes were assumed to be 2.5 percent of body weight in grams. If a body weight from around the time of the exam was not available, the most recent weight prior to the exam was used to calculate normal volumes. MN were then calculated by dividing measured volumes from calculated normal volumes. For the purpose of this manuscript, splenomegaly was defined as ≥2.0 MN.

Study data were in part collected and managed using REDCap (Research Electronic Data Capture) version 12.4.19 electronic data capture tools hosted at Duke University [28,29]. REDCap is a secure, web-based software platform designed to support data capture for research studies, providing (1) an intuitive interface for validated data capture; (2) audit trails for tracking data manipulation and export procedures; (3) automated export procedures for seamless data downloads to common statistical packages; and (4) procedures for data integration and interoperability with external sources.

Additional clinical data were pulled from DUHS electronic medical record, MaestroCare, and the International Collaborative Gaucher Group (ICGG) Registry (clinicaltrials.gov (last accessed on 26 October 2022)). In the case of discrepant data between the ICGG registry and MaestroCare, preference was given to information available in the latter.

### 2.2. Results

#### 2.2.1. Study Cohort

Longitudinal analysis of the clinical assessments including physical examination, treatment history, spleen and liver volumes, platelet counts, and biomarker analysis of lyso-Gb_1_ and CHITO was performed for 6 patients with type 1 GD and one patient with type 3 GD (Table 1).

#### 2.2.2. Clinical Parameters Improve and CHITO Activity Normalizes with Treatment

**Figure 1:**
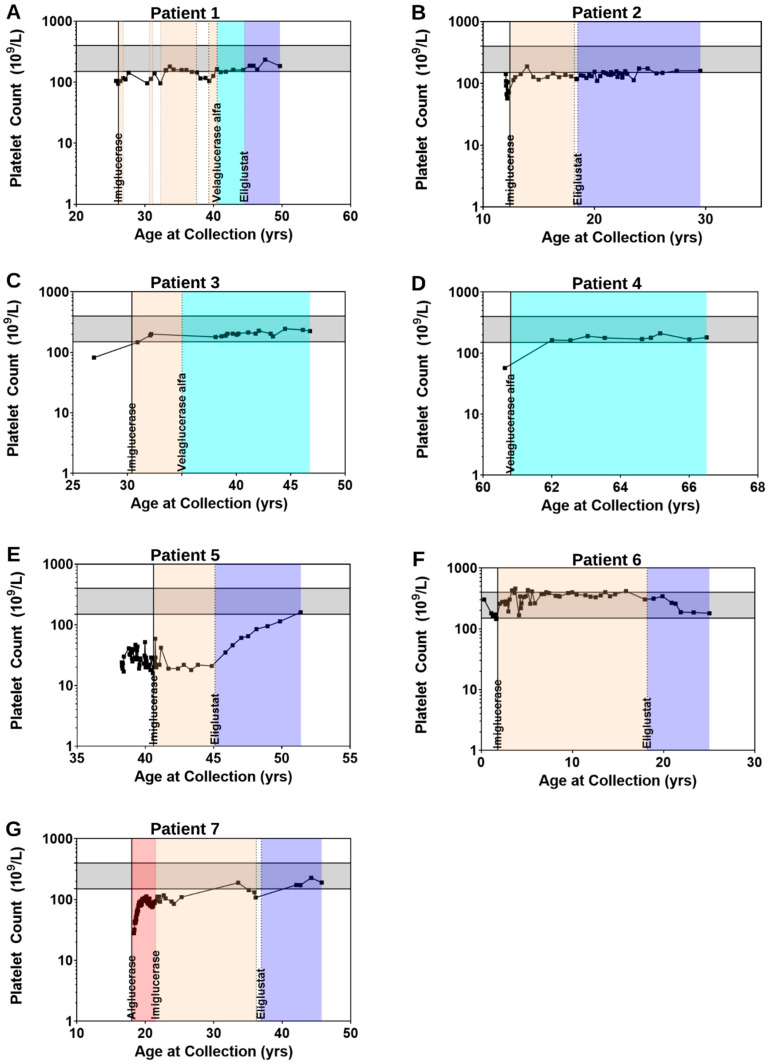
Platelet counts in (**A**–**G**) Patients 1–7, respectively. Vertical solid line denotes start of treatment and vertical dashed lines denotes either treatment switch or stop in treatment. Tan shading denotes treatment with imiglucerase, blue shading denotes treatment with velaglucerase alfa, purple shading denotes treatment with eliglustat, and red shading denotes treatment with alglucerase.

Patient 1 is a Caucasian male with type 1 GD who presented at the DUMC Metabolic Genetics clinic at age 30.3 years. He was diagnosed at age 25.9 years after a clinical history of joint pain, easy bruising, fatigue, splenomegaly (Supplemental Table S1), thrombocytopenia (105 × 10^9^ cells/L; Figure 1A) and a positive family history of GD prompted enzyme testing and genetic testing. Genetic testing revealed homozygous c.1226A>G; p.Asn409Ser pathogenic variants and confirmed a diagnosis of GD. At 26.1 years of age, ERT was initiated with imiglucerase 60 units/kilogram (U/kg) every two weeks until age 26.8 years after which he remained off of treatment.

**Figure 2:**
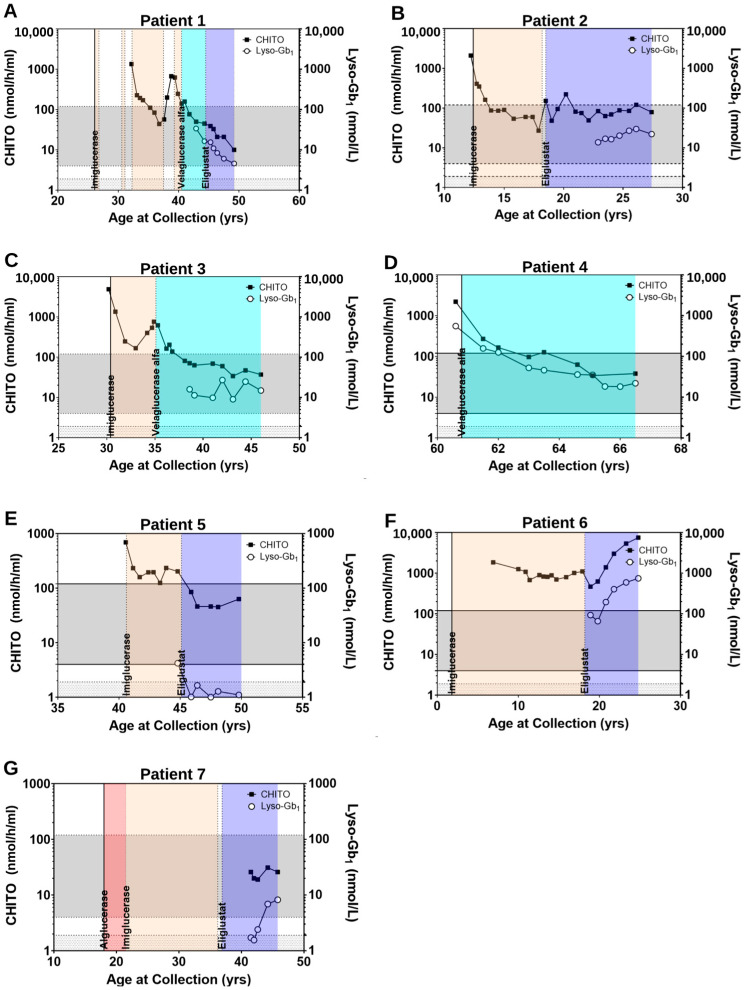
Biomarker values in (**A**–**G**) Patients 1–7, respectively. Vertical solid line denotes start of treatment and vertical dashed lines denotes either treatment switch or stop in treatment. Tan shading denotes treatment with imiglucerase, blue shading denotes treatment with velaglucerase alfa, purple shading denotes treatment with eliglustat, and red shading denotes treatment with alglucerase.

Upon initial examination at the DUMC Metabolic Genetics clinic at age 30.3 years, platelets remained low (98 × 10^9^ cells/L) and he was noted to have scattered bruising on anterior shins and noted mild occasional bony pain. Patient reinitiated treatment at age 30.7 years with imiglucerase; patient then experienced various dosage changes and multiple discontinuations through approximately age 40 years due to social factors including changes in insurance status. CHITO activity at age 32.2 years measured at 1347 nmol/h/mL) and continued a downward trend into the normal reference range on imiglucerase Figure 2A). During treatment discontinuation, CHITO activity increased to 673 nmol/h/mL. A similar response was observed with platelet counts (Figure 1A) and splenic volume in which during periods of treatment, improvements were noted. Due to global imiglucerase shortages [30], he switched to velaglucerase alfa at a dose of approximately 40 U/kg every two weeks as of age 40.5 years and CHITO levels responded by falling back into the normal reference range (average: 57.3 nmol/h/mL, range: 45–77).

At age 44.6 years, Patient 1 switched from velaglucerase alfa to eliglustat at a dose of 84 mg BID. During treatment with SRT, splenomegaly continued to improve, decreasing from 4.92 MN pre-treatment to 1.34 MN after approximately 5 years on eliglustat. Platelet counts continued to measure within the normal range and CHITO activity continued to decline on a downward trend (average: 24.8 nmol/h/mL, range: 10–39). This downward trend of CHITO activity was mirrored by the downward trend of lyso-Gb_1_ levels (Figure 2A), of note lyso-Gb_1_ levels were not measured during treatment with imiglucerase; however, continue to decrease, but have not normalized on treatment with velaglucerase alfa (average: 25.2 nmol/L, range: 16.4–33.9) or eliglustat (average: 9.1 nmol/L, range: 4.6–15.5). Upon evaluation at age 50.6 years, he was reported to be clinically stable with minimal symptoms of GD, denying any joint pain or increased bruising.

Patient 2 is a 30-year-old Caucasian male with type 1 GD who presented to the DUMC Metabolic Genetics clinic at 12.3 years of age. At age 12 years, he presented with pancytopenia, splenomegaly, and episodic epistaxis. Bone marrow biopsy and enzyme testing confirmed a diagnosis of GD. Genetic testing revealed homozygous c.1226A>G; p.Asn409Ser pathogenic variants. Dual energy X-ray absorptiometry (DEXA) scan performed following initial evaluation revealed osteopenia in the L1–L4 lumbar vertebrae with a Z score of −2.93. He began receiving treatment of GD with imiglucerase 60 U/kg every 2 weeks at age 12.4 years and subsequent dosing while on ERT largely remained between 52–60 U/kg every 2 weeks.

Prior to treatment, CHITO activity measured at 2105 nmol/h/mL. With treatment, CHITO activity declined and entered into the normal reference range after 1.5 years on treatment, and measured at its lowest after 5.5 years of ERT (average: 138.3 nmol/h/mL, range: 27–408; Figure 2B). In general, during treatment with imiglucerase, platelets largely remained low with baseline lab prior to treatment measuring at 72 × 10^9^ cells/L with pre-treatment values ranging from: 57–145 (Figure 1B). The patient was noted to have consistent reduction in splenic size from 13 MN to 3.4 MN after 5.5 years of treatment (Supplemental Table S1) and was clinically well without bone pain, bone crises, or increased fatigue.

At age 18.5 years, treatment switched to eliglustat at a dose of 84 mg once daily. Baseline CHITO activity prior to transition was borderline increased (152 nmol/hmL), that is most likely attributed to a two-month period of non-treatment prior to treatment switch. During the nearly 9 years of treatment with eliglustat, CHITO activity largely measured within reference limits (average: 93.7 nmol/h/mL, range: 48–223). Improvements in platelet counts were noted with values consistently measuring within the normal reference range starting approximately 5.5 years after transition to eliglustat. Lyso-Gb_1_ levels, which were only obtained during treatment with eliglustat, have remained stably elevated (average: 20.9 nmol/L, range: 13.8–29.8; Figure 2B) and stable mild splenomegaly persists.

Patient 3 is a Caucasian male with type 1 GD who presented to the DUMC Metabolic Genetics clinic at age 38.1 years. He presented at age 5 years with an enlarged spleen. Bone marrow biopsy performed at age 9 years revealed findings consistent with GD. Genetic testing revealed compound heterozygous c.1226A>G; p.Asn409Ser/c.475C>T; p.Arg159Trp pathogenic variants. Treatment with imiglucerase 45 U/kg every two weeks was initiated around 30.4 years of age, despite persistent thrombocytopenia in young adulthood, with a platelet count of 82 × 10^9^ cells/L at age 26.9 years (Figure 1C). Platelet counts normalized approximately 5 months after treatment initiation and there was a reduction of splenic size from 7.43 MN to 3.85 MN (Supplemental Table S1). Baseline CHITO activity measured at 4861 nmol/h/mL and decreased to 167 nmol/h/mL after approximately 2.6 years of treatment, though they began to uptrend over the next 1.9 years. During treatment with imiglucerase, the mean CHITO activity was 567.7 nmol/h/mL and values ranged from 167–1348.

DEXA scan performed approximately 2.7 years after treatment with imiglucerase was significant for mild osteopenia in the spine (Z-score: −1.1). After a reaction to imiglucerase during infusions (back pain and flank pain), the patient switched to treatment with velaglucerase alfa 45 U/kg every two weeks between ages 35 and 36 years and was reduced around age 39.4 year to 30 U/kg every 2 weeks given clinical stability. Following the switch to velaglucerase alfa, CHITO levels again began to downtrend, entering the normal range after approximately 3 years at age 38.1 years (average: 132.1 nmol/h/mL, range: 34–270; Figure 2C). Platelet counts remained within reference limits. Repeat DEXA scan after transition to velaglucerase alfa continued to show stable bone disease with persistent osteopenia of the spine. Spleen volume continued to down trend reaching its lowest size approximately 9.5 years after initiation of treatment with velaglucerase alfa, measuring 2.0 MN at age 44.5 years. Despite normalized CHITO activity, lyso-Gb_1_ levels, which were only obtained during treatment with velaglucerase alfa, remained elevated (average: 16.1 nmol/L, range: 9.0–27.0; Figure 2C) in the setting of stable, but mild disease burden as evidenced by persistent mild splenomegaly.

Patient 4 is a Caucasian male with type 1 GD who presented to the DUMC Metabolic Genetics clinic at age 60.6 years. Bone marrow biopsy was performed around age 60 years due to worsening pancytopenia and a history of easy bruising, fatigue, and bone pain which revealed findings consistent with GD. Genetic testing revealed homozygous c.1226A>G; p.Asn409Ser pathogenic variants, confirming the diagnosis. A DEXA scan was performed before diagnosis for a history of wrist, hip, and vertebral fractures which revealed decreased bone density (Z score of −2.3 for L1–L4 spine and −1.3 in the left proximal femur). Review of past medical records revealed thrombocytopenia (~80 × 10^9^ cells/L) and splenomegaly at age 40 years. At approximately age 60.8 years, treatment with velaglucerase alfa 60 U/kg every two weeks was initiated. Baseline labs prior to treatment revealed thrombocytopenia (57 × 10^9^ cells/L; Figure 1D), elevated CHITO activity (2205 nmol/h/mL), and elevated lyso-Gb_1_ (557.23 nmol/L; Figure 2D). While platelets normalized approximately 1 year after treatment initiation at age 62 years, CHITO activity which was elevated at baseline normalized approximately two years after treatment initiation (average: 113.4 nmol/h/mL, range: 34–270). During this time, lyso-Gb_1_ levels have continued to decline, yet remained elevated after nearly six years of treatment (average: 56.9 nmol/L, range: 18.2–157.3). Patient 4 continued to display mild splenomegaly (2.9 MN at age 64.9; Supplemental Table S1) and DEXA scan at age 65.7 noted stable bone density (Z scores for total spine was −2.4, total right femur was −2.2, right femoral neck was −1.6, and left proximal radius was −1.5). Upon clinical evaluation at 66 years of age, the patient was noted to have stable disease without complaints or evidence of bone pain, bone crises, or increased bruising or bleeding and ERT dosage was subsequently decreased to 45 U/kg every two weeks.

#### 2.2.3. Clinical Parameters Improve and Biomarker Levels Normalize upon Switch from ERT to SRT

Patient 5 is a Caucasian female of Ashkenazi Jewish descent with type 1 GD who presented to the DUMC Metabolic Genetics clinic at age 40.6 years. As previously reported in the literature, at age 33 years, she presented with thrombocytopenia, thought to be idiopathic; however, platelet counts did not respond to steroid therapy [31]. Bone marrow biopsy performed at approximately age 39 years was consistent with GD. Genetic testing revealed homozygous c.1226A>G; p.Asn409Ser pathogenic variants, confirming the diagnosis of GD. Upon establishing at Duke, imiglucerase treatment was reinitiated at a dose of 30 U/kg weekly to see if platelet counts would improve. There was no clinical benefit and other causes of thrombocytopenia were excluded. By age 41 years, the dose was then increased to 60 U/kg weekly. There was persistent thrombocytopenia throughout the period of uptitration which averaged 26 × 10^9^ cells/L and ranged from 16 to 59 × 10^9^ cells/L. She continued receiving weekly infusions through age 45.6 years. Throughout roughly 6 years of treatment with ERT, she continued to have persistent thrombocytopenia (Figure 1E), splenomegaly (Supplemental Table S1), elevated CHITO activity (average: 254.6 nmol/h/mL, range: 125–692), and elevated lyso-Gb_1_ (4.2 nmol/L; Figure 2E). She also had episodes of recurrent bruising, hematuria, and had complaints of fatigue.

Due to persistent thrombocytopenia and ongoing disease burden, at age 45.6 years, she began treatment with eliglustat. She was dually treated with ERT at a dose of 60 U/kg weekly and eliglustat 84 mg BID for approximately two months starting at age 45.6 years after which she switched to eliglustat 84 mg BID exclusively. Approximately 2–3 months after initiation of SRT, normalization of both CHITO activity and lyso-Gb_1_ levels was achieved for the first time (Figure 2E) with values remaining within reference limits throughout eliglustat treatment (CHITO average: 57 nmol/h/mL, range: 45–85; lyso-Gb_1_ average: 1.2 nmol/L, range: 1.0–1.7), and patient has since noted improvements in splenic volume (8 MN at age 47.5 years compared to 16.5 MN at age 40.7 years; Supplemental Table S1). Most strikingly, after six years of eliglustat treatment, platelet counts measured within reference limits (161 × 10^9^ cells/L at age 51.4 years; Figure 1E) for the first time in at least 13 years. Clinically, the patient self-reported no bruising and increased energy following SRT initiation.

#### 2.2.4. Clinical Parameters and Biomarker Levels Worsening upon Transition to SRT: Situations of Non-Compliance

Patient 6 is a Caucasian male with type 3 GD who presented to the DUMC Metabolic Genetics clinic at age 4.9 years. He was diagnosed at approximately 20 months after presenting with failure to thrive, anemia, neutropenia, and hepatosplenomegaly starting at age 18 months. Genetic testing revealed homozygous c.1448T>C; p.Leu483Pro pathogenic variants, confirming the diagnosis of GD. Treatment with imiglucerase 35 U/kg every two weeks was initiated at age 22 months with variable dosing between 35 and 48 U/kg every two weeks until age 4.9 years. Upon initial evaluation at Duke at age 4.9 years, anemia and neutropenia were noted to have resolved, but mild splenomegaly persisted (3.8 MN; Supplemental Table S1). He was referred for neurological evaluation and was noted to have vertical and horizontal saccadic eye movements. Given the diagnosis of type 3 GD the dose of ERT was eventually increased to 60 U/kg every two weeks.

Throughout treatment with ERT, platelet counts have consistently measured within the normal reference range (Figure 1F) whereas CHITO activity remained persistently elevated, despite treatment (average: 994.5 nmol/h/mL, range: 680–1852). At age 18 years splenomegaly had reached its lowest point throughout treatment on ERT measuring 2.2 MN (Supplemental Table S1) and pelvic MRI revealed bone involvement with a silent avascular necrosis of the femoral head.

Due to a history of significant needle phobia, patient switched to treatment with eliglustat 84 mg BID at 18.2 years of age. Pelvic MRI at approximately age 20 years showed resolution of the avascular necrosis of femoral head; but noted subcentimeter T2 enhancing stable lesions in the proximal bilateral femurs. CHITO activity decreased from 1107 nmol/h/mL to 467 nmol/h/mL after approximately 8 months of treatment with SRT; however, approximately one year later, CHITO levels began to trend upward and continued to rise above imiglucerase-treated levels (average: 3060 nmol/h/mL, range: 467–7515). Further evaluation revealed both self-reported poor compliance from the patient (missing one to three doses weekly) and clinical evidence of poor compliance with increasing lyso-Gb_1_ levels (average: 350.3 nmol/L, range: 66.1–747.2) in concordance with CHITO activity (Figure 2F) and in conjunction with worsening splenic and hepatic volumes on MRI at age 25 years (Supplemental Table S1).

Patient 7 is a Caucasian female with type 1 GD who presented to the DUMC Metabolic Genetics clinic at age 41.5 years. While splenomegaly was noted at age 5 years, a diagnosis of GD was not made until significant thrombocytopenia (27 × 10^9^ cells/L; Figure 1G) noted at age 17 years prompted a bone marrow biopsy with findings consistent with GD. Genetic testing revealed homozygous c.1226A>G; p.Asn409Ser pathogenic variants, confirming the diagnosis of GD. At approximately age 18.2 years, she began receiving treatment with alglucerase 60 U/kg every 2 weeks and transitioned to imiglucerase at variable dosing between ages 20 and 22 years. She continued imiglucerase at variable dosing until her last infusion at age 36.2, at which point she stopped treatment due to imiglucerase shortages. While on ERT, patient generally remained thrombocytopenic (Figure 1G) with mild improvements in splenic volume (16 MN at age 18.4 compared to 4.7 MN at age 36.1; Supplemental Table S1).

After receiving her last infusion at age 36.2 years, she experienced a 7-month period without treatment prior to switching to eliglustat 84 mg once daily at age 36.9 years. Eliglustat was reduced to 42 mg once daily around age 41.1 years for a five-month period due to elevated blood eliglustat levels, after which she returned to 84 mg once daily dosing. Upon transition to eliglustat, platelet counts, which were largely below normal while on ERT, normalized (Figure 1G) and splenic volume continued on a downward trend measuring 1.9 MN almost 10 years after treatment with eliglustat (Supplemental Table S1). Concerns surrounding compliance arose on evaluation at age 42.6 years, approximately 5.7 years after eliglustat initiation, when patient self-reported missed doses. At age 44.2, she self-reported stopping medication for two weeks immediately prior to evaluation. Upon clinical examination at 45.8 years of age, she reported taking medication approximately every other day, equating to 50% compliance with her daily dosing. While CHITO activity has remained within reference limits while on eliglustat (average: 24.4 nmol/h/mL, range: 19–31), lyso-Gb_1_ levels which initially measured within the normal reference range, have increased and are mildly elevated (average: 4.2 nmol/L, range: 19–31; Figure 2G), consistent with the self-reported non-compliance. Despite poor compliance and elevated lyso-Gb_1_, clinical reports indicate largely stable disease course as evidenced by decreased splenic volume, normal platelet count and DEXA scan at age 46.3 years, which revealed normal bone mineral density.

## 3. Discussion

The phenotypic heterogeneity of GD presents many challenges in patient diagnosis and management [32]. While the clinical benefits of treatment with ERT and/or SRT have been well documented in mouse models [33] and treatment naïve patients [13,34,35,36,37], considerable variation in the clinical response to treatment exists [32]. In one study, a comparison of lyso-Gb_1_ levels in patients on long-term ERT showed considerable inter- and intra-individual variability with higher concentrations of lyso-Gb_1_ observed in patients treated with imiglucerase in comparison to velaglucerase alfa [14] and with a steeper and faster decrease of lyso-Gb_1_ levels in those treated with velaglucerase alfa [18]. In a subsequent study, comparison of patients treated with ERT (imiglucerase and velaglucerase alfa) was similar and with SRT, a better clinical response was observed secondary to treatment with eliglustat [22,36]. The therapeutic effects of eliglustat were also demonstrated in a mouse model of GD, which showed notable decrease in lyso-Gb_1_ levels and improved clinical outcomes with earlier treatment initiation [33]. Due to the numerous treatment options available for GD and variable clinical and biochemical response to treatment observed, appropriate patient monitoring that includes lyso-Gb_1_ analysis should be in place to optimize individual treatment outcomes and treatment regimens.

Current guidelines for monitoring pediatric patients with type 1 GD recommend clinical evaluation performed at baseline should at a minimum include a physical examination, assessment of hematological, visceral, and skeletal organ systems, biomarker analysis with a focus on lyso-Gb_1_, and a questionnaire on pain and quality of life with follow-up of all areas aside from skeletal involvement every 6–12 months, and monitoring bone involvement every 12–24 months by MR or 24–36 months by DEXA. Furthermore, a change in treatment should warrant a physical examination, complete blood count (hemoglobin and platelets), and lyso-Gb_1_ analysis. For pediatric patients with type 3 GD, additional neurological and ophthalmological investigations are recommended [32]. The initial assessment and monitoring guidelines for adults with type 1 GD are in line with the aforementioned pediatric recommendations aside from biomarker monitoring which have not been update to include lyso-Gb_1_ analysis; measurement of non-specific analytes CHITO, angiotensin converting enzyme, and tartrate resistant acid phosphatase are still recommended [23].

While CHITO activity has been shown to be a useful tool for the diagnosis and monitoring of patients with GD, its analysis is unreliable in patients heterozygous or homozygous for the commonly inherited dup24 *CHIT1* variant as well as other documented rare pathogenic variants [38]. Patients with type 1 GD homozygous for the dup24 variant or compound heterozygous for the dup24 variant in trans with another pathogenic variant show no detectable plasma CHITO activity and carriers of the dup24 variant show an approximate 50% reduction of CHITO activity in comparison to wild-type [38,39]. These findings further support the utility of lyso-Gb_1_ for GD patient monitoring as it is a direct measure of substrate accumulation that has been shown to reflect therapeutic response [40].

Here, we present lessons learned on the clinical utility of lyso-Gb_1_ monitoring in seven patients with GD. (1) Lyso-Gb_1_ analysis was shown to be a more informative biomarker for patient monitoring, especially in the setting of normalized CHITO values; (2) clinical or biochemical improvements are observed in switch patients (ERT to SRT, or ERT to ERT); and (3) lyso-Gb_1_ should be used as tool to monitor compliance, specifically in eliglustat-treated patients.

Lyso-Gb_1_ has established itself as the most sensitive and specific GD biomarker with clinical utility in monitoring patients on treatment, reigning superior to CHITO activity. We demonstrate the clinical utility of lyso-Gb_1_ analysis in four patients with normal CHITO activity after treatment.

Patient 1 provides a unique, longitudinal example of a multiple switch-treatment patient and the benefits of switch treatment as determined by CHITO activity, lyso-Gb_1_ levels, and disease burden. During treatment with imiglucerase, platelet counts normalized and CHITO levels decreased, normalizing after approximately 3 years, but quickly rising above normal limits when treatment was interrupted. Similarly, a drop in platelet counts were noted during periods of non-treatment. After the first switch to velaglucerase alfa, platelet counts once again normalized and CHITO levels, which were already on a downward trend, further decreased normalizing 9.6 months after transition. In contrast, lyso-Gb_1_ concentrations, which were first assessed during treatment with velaglucerase alfa were modestly elevated. Due to the persistent elevations of lyso-Gb_1_ and splenomegaly, after four years, a second treatment switch to eliglustat occurred. Platelet counts, which were hovering at the lower limits of the normal reference range on ERT, continued to increase. CHITO activity, which was already normal, continued on a downward trend that was mirrored by a similar downward trend in lyso-Gb_1_ levels which were mildly elevated. Furthermore, after just over 3 years of SRT, his splenomegaly resolved. In following this patient’s biomarker values over time, it was evident that the normal CHITO activity during treatment were deceptive as there was ongoing underlying disease burden. The persistent elevations of lyso-Gb_1_ appear to be a more accurate reflection of the ongoing disease burden with levels declining but not yet plateauing.

Patients 2 and 3 further substantiate the clinical utility of lyso-Gb_1_ analysis in GD patients. Upon treatment with imiglucerase, CHITO activity in patient 2 normalized within 1.5 years; however, due to persistent thrombocytopenia and splenomegaly and in discussion with the patient, treatment switched to eliglustat. The patient responded with normalizing platelet counts and CHITO activity, and further reduction in splenic volume; however, lyso-Gb_1_ levels remained modestly elevated; however, appear to have reached a plateau. Platelet counts for patient 3 normalized after 8 months of treatment with imiglucerase; however, CHITO activity remained elevated throughout the 5 years of imiglucerase treatment. Patient 3 switched ERT treatment to velaglucerase alfa and CHITO activity normalized after 3 years. In contrast, lyso-Gb_1_ levels remain modestly elevated after 10 years of treatment with velaglucerase alfa and splenic volume was borderline, measuring in the high-normal range. For these patients, lyso-Gb_1_ levels while elevated, have settled into a ‘new-normal’ baseline for these patients and are a better reflection of the ongoing disease burden. These findings are consistent with previously published results that observed biomarker improvement or stability after ERT switch [18,41]; however, it should be noted that many other factors including dosing of ERT and periods of discontinuation, among others, may be contributing to this improvement following switch.

Patient 4 provides a unique perspective in these vignettes as the only patient for which untreated baseline levels of both lyso-Gb_1_ and CHITO activity were obtained. CHITO levels and platelet counts normalized approximately 2.5 years of treatment with velaglucerase alfa; whereas, lyso-Gb_1_ levels which were markedly elevated at baseline, were moderately elevated 2.5 years after ERT. Despite normalized platelets and CHITO activity, splenomegaly and osteoporosis/osteopenia persist in the setting of modestly elevated lyso-Gb_1_ highlighting the utility of lyso-Gb_1_ to more accurately reflect this patient’s ongoing disease burden.

These four patients leave us questioning the clinical utility of CHITO analysis as normal CHITO values may mask underlying residual disease burden. In a clinical situation, these values could contribute to treatment decisions that hinder disease stabilization; if normalized CHITO values are equated to stable disease, medication choice and dosing may remain stagnant when the patient could in fact benefit from a dose increase or medication switch. Lyso-Gb_1_ in contrast, remained elevated in these patients, reflecting the ongoing disease burden as evidenced by other markers of disease including organ volume, platelet counts, and/or bone density. Though longitudinal comparison of lyso-Gb_1_ to CHITO is difficult given the more recent recognition and utilization of lyso-Gb_1_ as a clinical biomarker, the evidence presented here clearly supports the utility of lyso-Gb_1_ as a longitudinal biomarker of disease burden. Furthermore, over the last several years, lyso-Gb_1_ biomarker analysis has been incorporated in the monitoring of large GD patient datasets from clinical trials as a reliable outcome measure for treatment efficacy and safety. In early 2020, the Food and Drug Administration (FDA) issued guidance for industry sponsored drug development and dose selection which encouraged the use of disease-specific biomarkers that reflect the underlying disease pathophysiology. As lyso-Gb_1_ is a direct reflection of substrate accumulation, it is evident that analysis of this biomarker should be included in the clinical monitoring of all patients with GD.

In contrast to the first four cases, patient 5 had both levels of lyso-Gb_1_ and CHITO activity fall into the normal range approximately 10.8 months after switching from imiglucerase to eliglustat. However, despite these values falling within normal ranges, she continued to have evidence of ongoing disease with persistent splenomegaly and significant thrombocytopenia. While she had modest improvements in her splenic volume, platelet counts, which did not respond to ERT, began a gradual incline for nearly 6 years before reaching the normal range. This patient highlights that in the setting of normal lyso-Gb_1_ ongoing disease burden may persist and thorough clinical evaluation is still needed.

Lyso-Gb_1_ is a particularly useful biomarker in tracking treatment compliance, particularly for patients on SRT with eliglustat. To demonstrate this, we presented two ERT to SRT switch patients, Patient 6 and Patient 7, who began exhibiting non-compliance. While dramatic elevations of lyso-Gb_1_ and CHITO activity were observed with missed doses in Patient 6, Patient 7 observed a mild elevation of lyso-Gb_1_, which was previously measuring within the normal reference range. In contrast, there was no concomitant increase in CHITO activity for Patient 7 as was observed in Patient 6 thus providing an increasingly compelling case regarding the utility of lyso-Gb_1_ for compliance monitoring.

We posit that lyso-Gb_1_ should be used as a tool in the clinical setting to monitor compliance. First, it has previously been published that changes in lyso-Gb_1_ levels often precede changes in other clinical biomarkers, including platelet counts and organomegaly [18,42]. Trending lyso-Gb_1_ levels in a monitoring plot may help clinicians identify potential cases of non-compliance before worsening of clinical symptoms and it may have clinical utility with regard to individualized therapeutic recommendations, allowing a conversation between patient and provider regarding which type of treatment would allow for the best compliance, and as a result, disease control. More individual case reports are needed to demonstrate the benefits of SRT in comparison to ERT with long-term lyso-Gb_1_ monitoring data. Furthermore, in view of the normalized CHITO levels in all of our patients with type 1 GD, integration of lyso-Gb_1_ would be a beneficial addition to the routine monitoring guidelines for adult patients with GD for treatment monitoring as well as to inform treatment decisions and other clinical determinations.

## 4. Conclusions

We describe seven patients with GD on long-term treatment with longitudinal clinical data and clinical monitoring based on current treatment guidelines. These clinical vignettes highlight the role of lyso-Gb_1_ as a sensitive biomarker in the management of patients with GD, and its particular utility when CHITO is normal and thus uninformative. We also describe the usefulness of lyso-Gb_1_ in tracking treatment non-compliance due to various factors. In doing so, we highlight the personalized medicine approach needed to optimize treatment and clinical outcomes for GD patients and highlight the need for further research including long-term clinical data at the individual level. 

## Figures and Tables

**Table 1:** Demographics.

Characteristic	Patients (*n* = 7)
Age at diagnosis in years, mean (range)	23.3 (1.5–60)
Sex, *n* (%)	
Male	5 (71)
Female	2 (29)
Race, *n* (%)	
Caucasian	7 (100)
Subtype, *n* (%)	
Type 1	6 (86)
Type 3	1 (14)
Genotype, *n* (%)	
p.Asn409Ser/p.Asn409Ser	5 (29)
p.Leu483Pro/p.Leu483Pro	1 (14)
p.Asn409Ser/p.Arg159Trp	1 (14)
Splenectomy, *n* (%)	0
Naïve & treatment status, *n* (%)	
Naïve	3 (43)
Treated non-naïve	4 (57)
Treatment type, *n* (%)	
ERT	1 (14)
ERT to ERT switch	1 (14)
ERT to ERT to SRT switch	1 (14)
ERT to SRT switch	4 (57)

## Data Availability

Data cannot be shared due to ethical and privacy issues.

## Supplementary Materials

The supporting information can be downloaded at: https://www.mdpi.com/article/10.3390/ijms232314938/s1.
