# Supplementary material for: Glucosylsphingosine (Lyso-Gb1): An Informative Biomarker in the Clinical Monitoring of Patients with Gaucher Disease
Source: Int J Mol Sci. 2022 Nov 29;23(23):14938. doi: 10.3390/ijms232314938 (PMC9736277; doi:10.3390/ijms232314938)
Supplement: Supplementary file 1 [file ijms-23-14938-s001.zip › ijms-2045756-supplementary.pdf]

Supplemental Table S1. Patient organ volumes.

		Spleen		Liver	
	**Age (yrs)**	**Volume (ml)**	**MN**	**Volume (ml)**	**MN**
Patient 1	30.3	769	4.9	2109	1.1
	31.4	643	3.8	1754	0.8
	33.1	821	4.8	1800	0.8
	34.8	406	2.3	1429	0.6
	36.8	459	2.8	1840	0.9
	38.1	428	2.7	1644	0.8
	39.9	364	2.2	1663	0.8
	41.0	368	2.2	1742	0.8
	44.3	**319**	**1.8**	1622	0.7
	46.4	**306**	**1.7**	1724	0.8
	49.7	**254**	**1.3**	1649	0.7
Patient 2	12.4	870	13.3	1098	1.3
	13.4	555	6.5	1097	1.0
	15.0	569	5.2	1119	0.8
	16.3	527	4.8	1163	0.8
	17.9	408	3.4	1183	0.8
	24.1	535	3.9	1497	0.9
	27.1	555	3.6	1474	0.8
Patient 3	30.2	1300	7.4	2600	1.2
	32.2	673	3.8	2200	1.0
	37.5	498	2.6	2186	0.9
	38.8	526	2.7	2543	1.0
	40.2	474	2.4	2294	0.9
	42.1	506	2.4	2594	1.0
	44.5	394	2.0	2182	0.9
	47.0	417	2.1	2557	1.0
Patient 4	62.1	382	3.1	1593	1.0
	64.9	345	2.9	1502	1.0
Patient 5	40.7	1906	16.5	1671	1.2
	41.7	1839	16.2	1851	1.3
	43.9	1632	13.7	1951	1.3
	44.9	1440	11.7	1605	1.0
	45.9	1494	12.5	1732	1.2
	47.5	954	8.0	1797	1.2
Patient 6	1.8	355	16.0	631	2.3
	2.8	125	4.6	476	1.4
	4.0	90	2.9	210	0.5
	4.9	130	3.8	595	1.4
	5.9	141	3.9	763	1.7
	6.6	134	3.3	622	1.2
	7.5	151	3.4	775	1.4
	11.4	168	2.8	978	1.3
	13.6	204	2.8	1001	1.1
	15.9	278	2.9	1372	1.2
	17.9	225	2.2	1149	0.9
	19.9	269	2.6	1285	1.0
	22.3	677	6.5	1804	1.4
	25.0	995	8.7	2208	1.5
Patient 7	18.4	2055	16.0	2286	1.4
	18.9	1288	9.7	2043	1.2
	19.4	1109	8.0	1947	1.1
	19.9	993	7.5	1859	1.1
	20.4	1000	7.3	2031	1.2
	20.9	900	6.8	2030	1.2
	22.0	751	5.8	2092	1.3
	23.0	694	5.3	1750	1.1
	24.2	580	4.7	1864	1.2
	36.1	592	4.5	1584	1.0
	41.1	363	2.7	1521	0.9
	44.3	315	2.2	1761	1.0
	46.3	**283**	**1.9**	1915	1.0

Gray shading denotes treatment switch to velaglucerase alfa.
Blue shading denotes treatment switch to eliglustat.
Bold numbers denotes normal splenic volume.
